# Evaluation of presumptive identification of *Enterobacterales* using CHROMagar Orientation medium and rapid biochemical tests

**DOI:** 10.1002/jcla.23453

**Published:** 2020-06-28

**Authors:** Hirofumi Ohtaki, Akifumi Takahashi, Ayumi Niwa, Jun Yonetamari, Asami Nakayama, Tomokazu Kuchibiro, Hirotoshi Ohta, Hiroyasu Ito, Hisashi Baba, Nobuo Murakami, Kiyofumi Ohkusu

**Affiliations:** ^1^ Department of Clinical Laboratory Science Graduate School of Kansai University of Health Sciences Osaka Japan; ^2^ Department of Informative Clinical Medicine Gifu University Graduate School of Medicine Gifu Japan; ^3^ Department of Clinical Laboratory Gifu University Hospital Gifu Japan; ^4^ Department of Clinical Laboratory Yamagata University Hospital Yamagata Japan; ^5^ Department of Clinical Laboratory Naga Municipal Hospital Kinokawa Japan; ^6^ Center for Nutrition Support & Infection Control Gifu University Hospital Gifu Japan; ^7^ Department of Microbiology Tokyo Medical University Tokyo Japan

**Keywords:** CHROMagar Orientation medium, *Enterobacterales*, presumptive identification, rapid biochemical tests

## Abstract

**Background:**

The use of matrix‐assisted laser desorption/ionization time‐of‐flight mass spectrometry is gradually spreading among large‐scale laboratories; however, this method is impractical for small‐scale laboratories. In laboratories without access to these rapid identification methods, problems related to them remain unsolved. In this study, we aimed to develop a rapid and inexpensive method to presumptively identify *Enterobacterales* using CHROMagar Orientation medium.

**Methods:**

The algorithm for presumptive identification of *Enterobacteriaceae* using CHROMagar Orientation medium was based on our previous studies. Modified property tests for indole, lysine decarboxylase, ornithine decarboxylase, and hydrogen sulfide were performed to evaluate the differentiation of the bacterial species.

**Results:**

Using the type strains and clinical isolates, it was possible to conduct the property tests at a low cost, within 4 hours. The spot indole test was performed without any nonspecific reactions for the bacteria forming colored colonies. The presumptive identification of bacteria was thereby possible within 24 hours after specimen submission.

**Conclusion:**

All these results suggest that the rapid presumptive identification of *Enterobacterales* is possible with this new identification method using CHROMagar Orientation medium. This is therefore a prompt and economical method that can be used in routine laboratory work.

## 1 INTRODUCTION

Rapid and accurate identification of bacteria is extremely important in clinical microbial laboratory testing. The use of matrix‐assisted laser desorption/ionization time‐of‐flight mass spectrometry (MALDI‐TOF MS) for rapid identification of bacteria has been gradually spreading in recent years and is becoming an increasingly available and widely used method.^1^, ^2^ In many clinical cases, the introduction of MALDI‐TOF MS has enabled the reporting of identification results the day after culture. In addition to the promotion of antimicrobial stewardship, the significance of rapid reporting has increased. Facilities that cannot acquire MALDI‐TOF MS equipment due to installation costs are still required to report identification results promptly. As an alternative, the use of simplified conventional tests to assess biochemical properties can provide these laboratories with rapid identification capabilities. The Clinical and Laboratory Standards Institute (CLSI) document M35‐A2 and other guidelines are resources that support the rapid identification of bacteria.^3^ In addition, the operation of routine work using selective medium is highly useful.^4^, ^5^ Our research group has previously proposed an algorithm for the presumptive identification of *Enterobacterales*, which are frequently isolated in the extraintestinal infection using the CHROMagar Orientation medium.^6^ This is an inexpensive and useful method that was developed by combining the colony color and property tests. However, the considerably long time required to perform the property tests for metallic blue and brown colonies is a disadvantage. To solve this problem, it is necessary to quickly determine bacterial properties using biochemical methods including the indole, lysine decarboxylase (LDC), ornithine decarboxylase (ODC), and hydrogen sulfide (H_2_S) tests. Therefore, the aim of our study is to establish a rapid method using this algorithm.

## 2 MATERIALS AND METHODS

### 2.1 Algorithm for presumptive identification

The algorithm for presumptive identification of *Enterobacterales* using CHROMagar Orientation medium was based on previous studies (Table 1). Briefly, this method interrogates properties required for each color tone of the colonies growing on the medium and performs presumptive identification by combining their results. In this study, four property tests (indole, LDC, ODC, and H_2_S) were performed to distinguish each bacterial species. Since brown color colonies already indicate indole pyruvic acid (IPA) production, the property of IPA was deleted in this algorithm. We conducted the following study so that the above property tests could be performed quickly and inexpensively.

**TABLE 1:** The algorithm for presumptive identification of *Enterobacterales* using CHROMagar Orientation medium

Colony color	IND	LDC	ODC	H_2_S	Expected isolate(s)
Purplish pink	+				*Escherichia coli*
Metallic blue	−	+	−		*Klebsiella pneumoniae*
	+	+	−		*Klebsiella oxytoca*
	−	+	+		*Klebsiella aerogenes*
	−	−	+		*Enterobacter cloacae*
	+ or −	−	−		*Citrobacter freundii*
	+	−	+		*Citrobacter koseri*
Aqua blue					*Serratia marcescens*
Brown	−	−	+	+	*Proteus mirabilis*
	−	−	−	+	*Proteus penneri*
	+	−	−	+	*Proteus vulgaris*
	+	−	−	−	*Providencia* sp.
	+	−	+	−	*Morganella morganii*
Other colors	Should be confirmed by other methods				

Abbreviations: −, negative reaction; +, positive reaction; IND, indole; LDC, lysine decarboxylase; ODC, ornithine decarboxylase.

### 2.2 Investigation of medium volume and incubation time in the LDC and ODC tests

The media for the LDC and ODC tests were prepared using Moeller decarboxylase broth base (Becton‐Dickinson), and each medium was adjusted to a 2% concentration using l‐lysine monohydrochloride (FUJIFILM Wako Pure Chemical Corporation) or l‐ornithine monohydrochloride (FUJIFILM Wako Pure Chemical Corporation). The LDC and ODC test media were prepared at liquid volumes of 0.1 mL (low‐volume method) and 3 mL (conventional method). For the low‐volume method, a 0.2 mL tube was used as a medium container. Bacteria adhered to the tip of an inoculating needle were inoculated into the medium (approximately 10^6^ CFU), and the results were determined at 2, 4, 6, 8, and 24 hours after incubation (35°C, aerobic condition). American Type Culture Collection (ATCC) strains (*Klebsiella pneumoniae* ATCC 13883 with LDC positive and ODC negative, *Enterobacter cloacae* ATCC 13047 with LDC negative and ODC positive, *Klebsiella aerogenes* ATCC 13048 with LDC positive and ODC positive) grown on CHROMagar Orientation medium (Kanto Chemical Co., Inc) were used for the examination. A sample that showed a purple colored medium was regarded as positive and judged by agreement of two medical technologists. In addition, the color of the medium was objectively evaluated by measuring the absorbance at 570 nm with a microplate reader. A value of 0.2 or more was determined as a visible purple color. Absorbance in 0.1 and 3 mL media containing lysine or ornithine was subject to statistical analysis. In each experiment, the results were expressed as mean ± SD. The statistical significance of the difference in mean values was determined by Student's *t* test. *P* values of less than .05 were considered significant.

### 2.3 Investigation of medium volume and incubation time in the hydrogen sulfide production test

The SIM medium for the H_2_S production test was prepared according to past literature.^7^ The composition of the medium was as follows: 0.3% beef extract (Nacalai Tesque, Inc), 3% peptone (Nacalai Tesque, Inc), 0.005% sodium thiosulfate (FUJIFILM Wako Pure Chemical Corporation), 0.02% L‐cysteine hydrochloride monohydrate (FUJIFILM Wako Pure Chemical Corporation), and 0.05% ferric ammonium citrate (FUJIFILM Wako Pure Chemical Corporation). The medium was prepared with and without agar (ie, semi‐solid agar and broth were prepared, respectively). Each medium was aliquoted at 0.1 and 3 mL for the low‐volume and conventional methods, respectively. For the 0.1 mL volume, a 0.2 mL tube was used as the medium container. Bacteria adhered to the tip of an inoculating needle were inoculated into the medium (approximately 10^6^ CFU), and the results were determined at 2, 4, 8, and 24 hours after incubation (35°C, aerobic condition). ATCC and NITE Biological Resource Center (NBRC) strains (*Proteus mirabilis* ATCC 7002 with H_2_S positive, *Proteus vulgaris* ATCC 13315 with H_2_S positive, and *Morganella morganii* NBRC 3168 with H_2_S negative) grown on CHROMagar Orientation medium were used for the examination. A sample that showed black colored medium was regarded as positive and judged by agreement of two medical technologists.

### 2.4 Evaluation of the accuracy of new identification methods using clinical isolates

Based on the algorithm (Table 1) and the results (Figures S1 and S2), a study using clinical isolates was conducted. Using 508 clinical strains (*Enterobacterales* with Oxidase negative) that produced metallic blue, aqua blue or brown colonies on CHROMagar Orientation medium isolated in Gifu University Hospital from 2014 to 2019, the accuracy of the new identification method was determined based on the results of MALDI Biotyper (Bruker Daltonics) under blinded conditions. These 508 strains included *K. pneumoniae* (n = 157), *Klebsiella oxytoca* (n = 41), *K. aerogenes* (n = 34), *Klebsiella variicola* (n = 25), *Raoutella ornithinolytica* (n = 2), *Raoultella planticola* (n = 3), *E. cloacae* (n = 85), *Citrobacter freundii* (n = 26), *Citrobacter koseri* (n = 16), *Citrobacter braakii* (n = 3), *Citrobacter amalonaticus* (n = 1), *Citrobacter farmer* (n = 1), *Serratia marcescens* (n = 26), *P. mirabilis* (n = 44), *Proteus penneri* (n = 5), *P. vulgalis* (n = 7), *Providencia* sp. (n = 10), *M. morganii* (n = 21), and *Pantoea* sp. (n = 1). For the identification of the metallic blue colony and the brown colony, the LDC, ODC, H_2_S, and spot indole tests were performed according to the algorithm (Table 1). The LDC test medium and ODC test medium were aliquoted at volumes of 0.1 mL. As a medium for the H_2_S production test, 0.1 mL of agar‐free medium was prepared. For the LDC, ODC, and H_2_S tests, bacteria adhered to the tip of a platinum needle were inoculated into the medium, and the results were determined at 4 hours after incubation (35°C, aerobic condition). In the spot indole test, reagents whose colony color did not affect the test results were selected. Therefore, Kovac's reagent was used for the metallic blue colonies and *p*‐dimethylaminocinnamaldehyde (DMACA) reagent was used for the brown colonies.

## 3 RESULTS

### 3.1 Effect of medium volume and incubation time on the LDC, ODC, and H_2_S tests

The LDC and ODC test results for three bacterial species of the ATCC strain could be determined 4 hours after inoculation using the low‐volume method even if they could not be determined by the conventional method (Figure S1). Four hours was chosen as the test time point because after 24 hours incubation, a positive reaction was also observed in the negative control of the low‐volume method and at 2 hours the positive reaction after incubation in the low‐volume method was too weak.

In the H_2_S‐producing strains *P. mirabilis* and *P. vulgaris*, blackening was observed 4 hours after inoculation in 0.1 mL of SIM medium without agar but not in the other media at the same incubation time (Figure S2). A positive reaction at 2 hours after culture was not observed in any sample (data not shown). The H_2_S‐non‐producing strain *M. morganii* showed negative reactions in all media. Therefore, it was decided that evaluation of H_2_S production can be judged in 0.1 mL of agar‐free SIM medium at 4 hours.

### 3.2 Evaluation of the accuracy of new identification methods using clinical isolates

The new identification method, which is a combination of the improved property tests (LDC, ODC, and H_2_S) and the spot indole test, was compared with the MALDI‐TOF MS method for identifying bacterial species forming blue and brown colonies on CHROMagar Orientation medium. The concordance rate based on the results of MALDI Biotyper was 90.9% at the species level and 96.3% at the genus level. In addition, when bacterial species that could not be identified by this algorithm were removed, the concordance rate was 97.8% the species level and 97.7% the genus level. For species not included in the algorithm, 75% of them could be identified at the genus level (Table 2). This method showed misidentification at a rate of 2.3% (n = 11) in the bacterial species included in the algorithm. Misidentification was caused by the lack of typical characteristics observed in these strains (Table 3). We confirmed that there is no difference between the results of the new method and the conventional method in these strains (data not shown).

**TABLE 2:** Evaluation of the new method using clinical isolates

Category	Species (number of strains)	Species level			Genus level		
		Concordance	Discordance	Accuracy	Concordance	Discordance	Accuracy
A. Bacterial species included in the algorithm	*Klebsiella pneumoniae* (157)	156	1	99.4%	156	1	99.4%
	*Klebsiella oxytoca* (41)	41	0	100%	41	0	100%
	*Klebsiella aerogenes* (34)	34	0	100%	34	0	100%
	*Enterobacter cloacae* (85)	82	3	96.5%	82	3	96.5%
	*Citrobacter freundii* (26)	25	1	96.2%	25	1	96.2%
	*Citrobacter koseri* (16)	16	0	100%	16	0	100%
	*Serratia marcescens* (26)	22	4	84.6%	22	4	84.6%
	*Proteus mirabilis* (44)	43	1	97.7%	43	1	97.7%
	*Proteus vulgaris* (7)	7	0	100%	7	0	100%
	*Proteus penneri* (5)	5	0	100%	5	0	100%
	*Morganella morganii* (21)	21	0	100%	21	0	100%
	*Providencia* sp. (10)	N/A	N/A	N/A	9	1	90.0%
	Total (472)	452	10	97.8%	461	11	97.7%
B. Bacterial species not included in the algorithm	*Klebsiella variicola* (25)	0	25	0%	25	0	100%
	*Raoultella ornithinolytica* (2)	0	2	0%	0	2	0%
	*Raoultella planticola* (3)	0	3	0%	0	3	0%
	*Citrobacter braakii* (3)	0	3	0%	1	2	66.7%
	*Citrobacter amalonaticus* (1)	0	1	0%	1	0	100%
	*Citrobacter farmeri* (1)	0	1	0%	1	0	100%
	*Pantoea* sp. (1)	N/A	N/A	N/A	0	1	0%
	Total (36)	0	35	0%	28	8	75.0%
A + B	Total (508)	452	45	90.9%	489	19	96.3%

Abbreviation: N/A, not applicable.

**TABLE 3:** Causes of misidentification in the new method

Category	Species (number of strains)	Identification results by the algorithm (number of strains)	The causes of discordance
Bacterial species included in the algorithm	*Klebsiella pneumoniae* (1)	*Citrobacter freundii* (1)	LDC negative
	*Enterobacter cloacae* (3)	*Citrobacter freundii* (2)	ODC negative
		*Klebsiella aerogenes* (1)	LDC positive
	*Citrobacter freundii* (1)	*Klebsiella pneumoniae* (1)	LDC positive
	*Serratia marcescens* (4)	*Klebsiella aerogenes* (4)	Colony color (metallic blue)
	*Proteus mirabilis* (1)	Not identified (1)	H_2_S negative
	*Providencia* sp. (1)	Not identified (1)	IND negative
Bacterial species not included in the algorithm	*Klebsiella variicola* (25)	*Klebsiella pneumoniae* (24)	Not included in the algorithm
		*Klebsiella oxytoca* (1)	
	*Raoultella ornithinolytica* (2)	*Klebsiella oxytoca* (1)	
		*Citrobacter freundii* (1)	
	*Raoultella planticola* (3)	*Klebsiella pneumoniae* (3)	
	*Citrobacter braakii* (3)	*Citrobacter freundii* (1)	
		*Enterobacter cloacae* (1)	
		Not identified (1)	
	*Citrobacter amalonaticus* (1)	*Citrobacter koseri* (1)	
	*Citrobacter farmeri* (1)	*Citrobacter koseri* (1)	
	*Pantoea* sp. (1)	Not identified (1)	

For the spot indole test, to avoid confusion between colony color and color tone indicating a positive result, the blue colony test was performed with Kovac's reagent and the brown colony test was performed with DMACA reagent. Nonspecific reactions were not observed for any strain, and it was therefore not included as a cause of misidentification (Figure S3; Table 3).

The ATCC and NRBC strains (*K. pneumoniae* ATCC 13883 with LDC positive and ODC negative, *E. cloacae* ATCC 13047 with LDC negative and ODC positive, *P. mirabilis* ATCC 7002 with H_2_S positive, and *M. morganii* NBRC 3168 with H_2_S negative) were used as a quality control for the property test media, and it was confirmed that the media were stable for 1 year under refrigeration (4°C) or freezing (−20°C).

**FIGURE 1:**
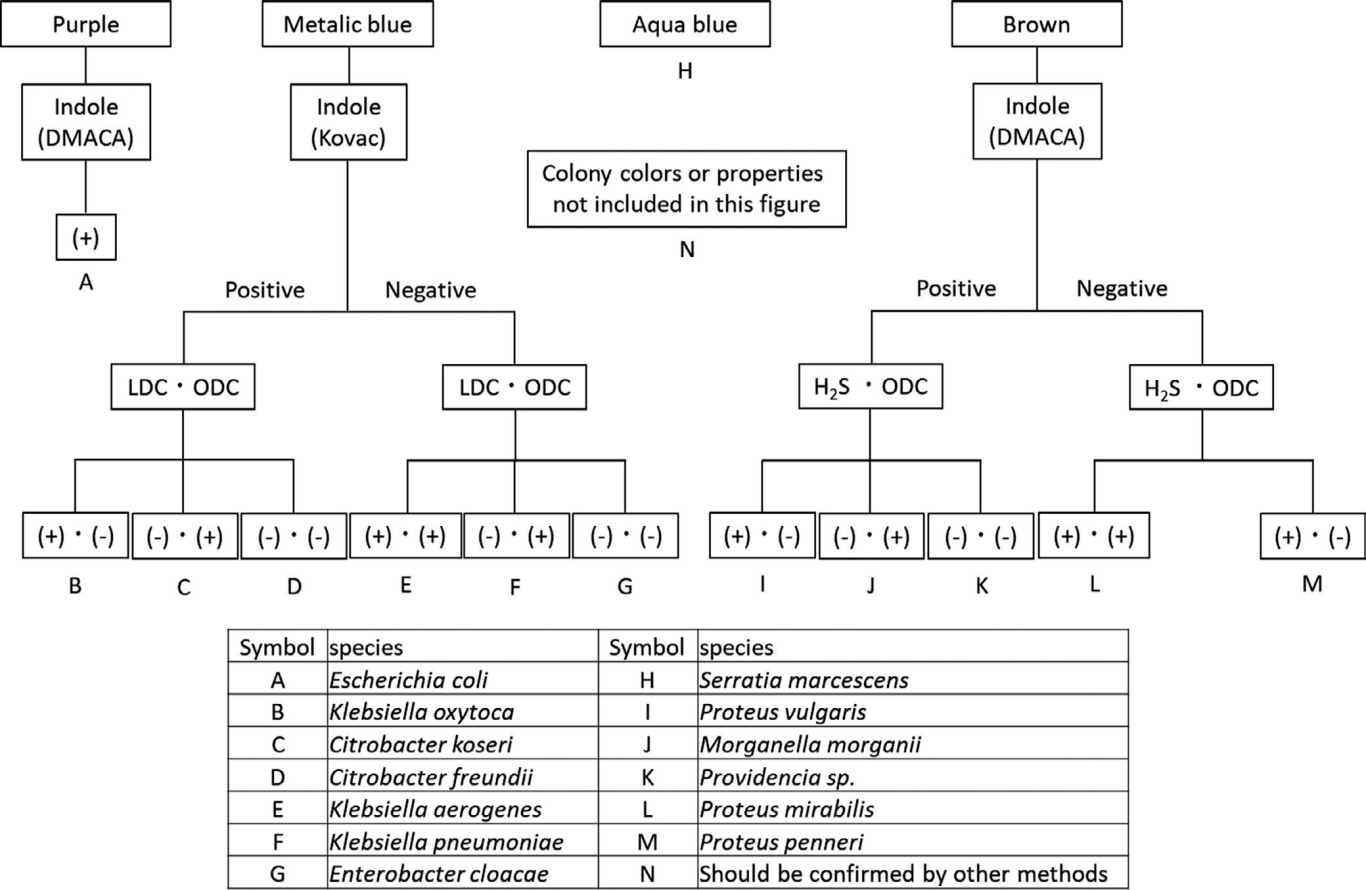
Flowchart of presumptive identification method using CHROMagar Orientation medium. DMACA, *p*‐dimethylaminocinnamaldehyde reagent; H_2_S, hydrogen sulfide; Kovac, Kovac's reagent; LDC, lysine decarboxylase; ODC, ornithine decarboxylase

The flowchart of this method based on these results and previous findings is shown in Figure 1.

## 4 DISCUSSION

The primary disadvantage of the conventional method of presumptive bacterial identification is that an entire day is required for the property tests using OIML and SIM media. Thus, results cannot be reported until the third day, which negatively impacts reporting speed. In this study, several investigations were carried out with the objective of shortening the identification reporting time. Our presumptive identification method using CHROMagar Orientation medium is a useful technique that can be easily performed at a low cost. It is useful in the presumptive identification of *Enterobacterales*, which are frequently isolated in extraintestinal infections, and is well adapted for the examination of specimens such as urine, sputum, pus, ascitic fluid, and blood. In addition, this algorithm has sufficient accuracy to be used as a presumptive identification method.^6^

The use of the LDC and ODC tests allowed for correct judgment at 4 hours in ATCC strains; it is necessary to keep this 4‐8 hours evaluation time point, as false‐positive reactions are observed after 24 hours. In the study of H_2_S productivity using ATCC strains, H_2_S production was visible at 4 hours in 0.1 mL of agar‐free SIM medium. In all these tests, it was possible to perform evaluations without issues, even in the study using clinical isolates. In the study using clinical isolates, 11 of the strains included in the algorithm could not be identified. These were strains that do not exhibit typical properties, highlighting a limit of this method. In particular, it should be noted that there are some *S. marcescens* that form metallic blue colonies. With the spot indole test, which is generally not recommended for colored colonies, it was possible to determine results without experiencing false‐positive or false‐negative reactions in any of the reagents. In other words, it was shown that the method using two types of spot indole reagents was useful for metallic blue and brown colonies grown in CHROMagar Orientation medium. Combinations consisting of a spot indole test as the first step and rapid property tests (LDC, ODC, and H_2_S) as the second step enable reporting of estimated results within 24 hours of culture initiation. This study suggests that this new method may be useful for the rapid presumptive identification of *Enterobacterales* in many cases.

CHROMagar Orientation medium is generally used with an antibacterial agent for the detection of extended‐spectrum β‐lactamase (ESBL)‐producing and carbapenemase‐producing bacteria in *Enterobacterales* infections.^8^ Using selective media and the new identification method discussed, it becomes possible to report on the presumed bacterial species and drug‐resistance factors within 24 hours of specimen submission. Considering that the detection rate of ESBL‐producing bacteria in Japan has risen compared to 20 years ago^9^ and that of carbapenemase‐producing bacteria is also increasing,^10^, ^11^ there is a significant need for the implementation of this method as a screening test. Due to the importance of antimicrobial stewardship in recent years,^12^ early presumptive reports on identification and resistance factors are extremely meaningful. Therefore, there are various opportunities for using this new method.

Furthermore, due to the spread of resistant bacteria, wide‐range MIC measurement beyond the vicinity of the break point is desired from the viewpoint of the treatment and detection of resistant bacteria. However, in many facilities, combiplate testing (which is the same plate test used for identification and drug susceptibility testing) is adopted due to their cost and operability. Cost reduction in the identification step is important because it leads to upgradation of the drug susceptibility test. Introduction of MALDI‐TOF MS helps to solve these problems, but in some small‐scale facilities, it may not be introduced due to high costs. It is presumed that the newly proposed method in this paper can be utilized effectively in such cases.

CLSI M35‐A2 is often cited as a manual for simplified identification methods, containing descriptions of methods for the identification of 37 bacterial species, most of which can be performed at low cost and in a short time frame. These methods are considered to be useful not only for convenience but also as alternative methods in unexpected cases such as disasters and machine failures. In order for this new method to be used as commonly as those described in this manual, we intend to continue our studies and provide more information in the future.

## CONFLICT OF INTEREST

The authors declare that they do not have any competing financial interests.

## Supporting information

Fig S1‐S3
