# Supplementary figures and images for: Evaluation of presumptive identification of Enterobacterales using CHROMagar Orientation medium and rapid biochemical tests
Source: J Clin Lab Anal. 2020 Jun 28;34(10):e23453. doi: 10.1002/jcla.23453 (PMC7595914; doi:10.1002/jcla.23453)

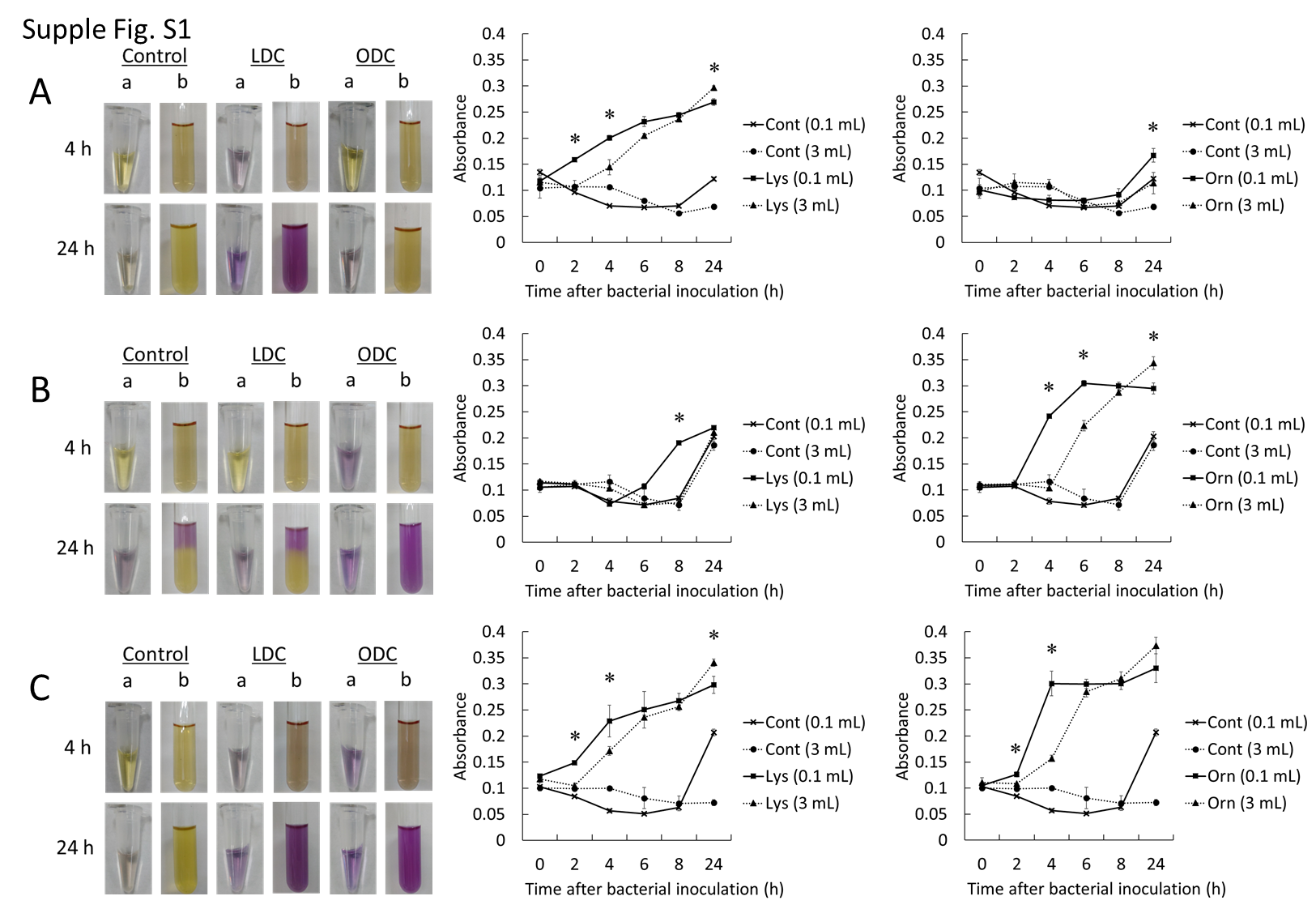


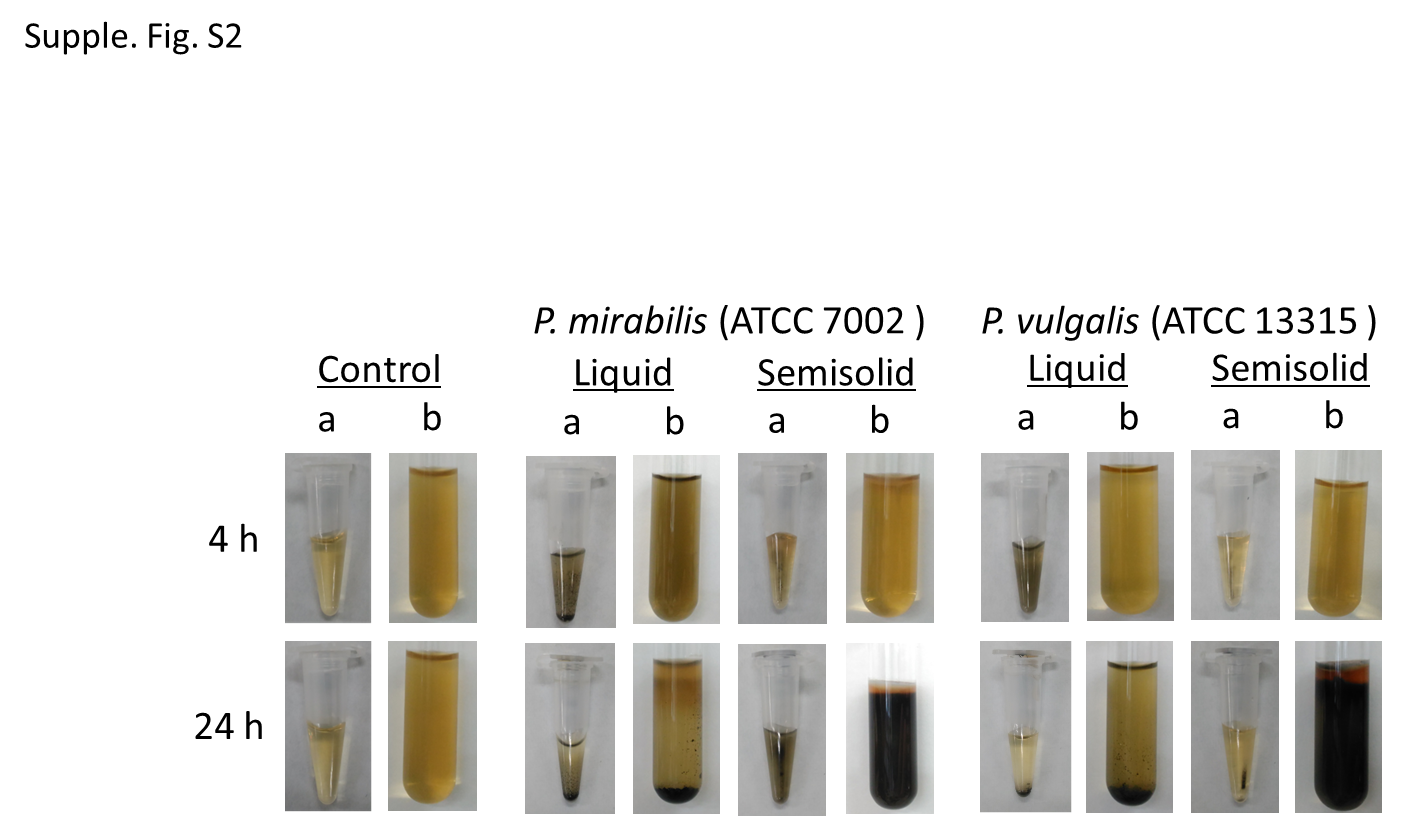


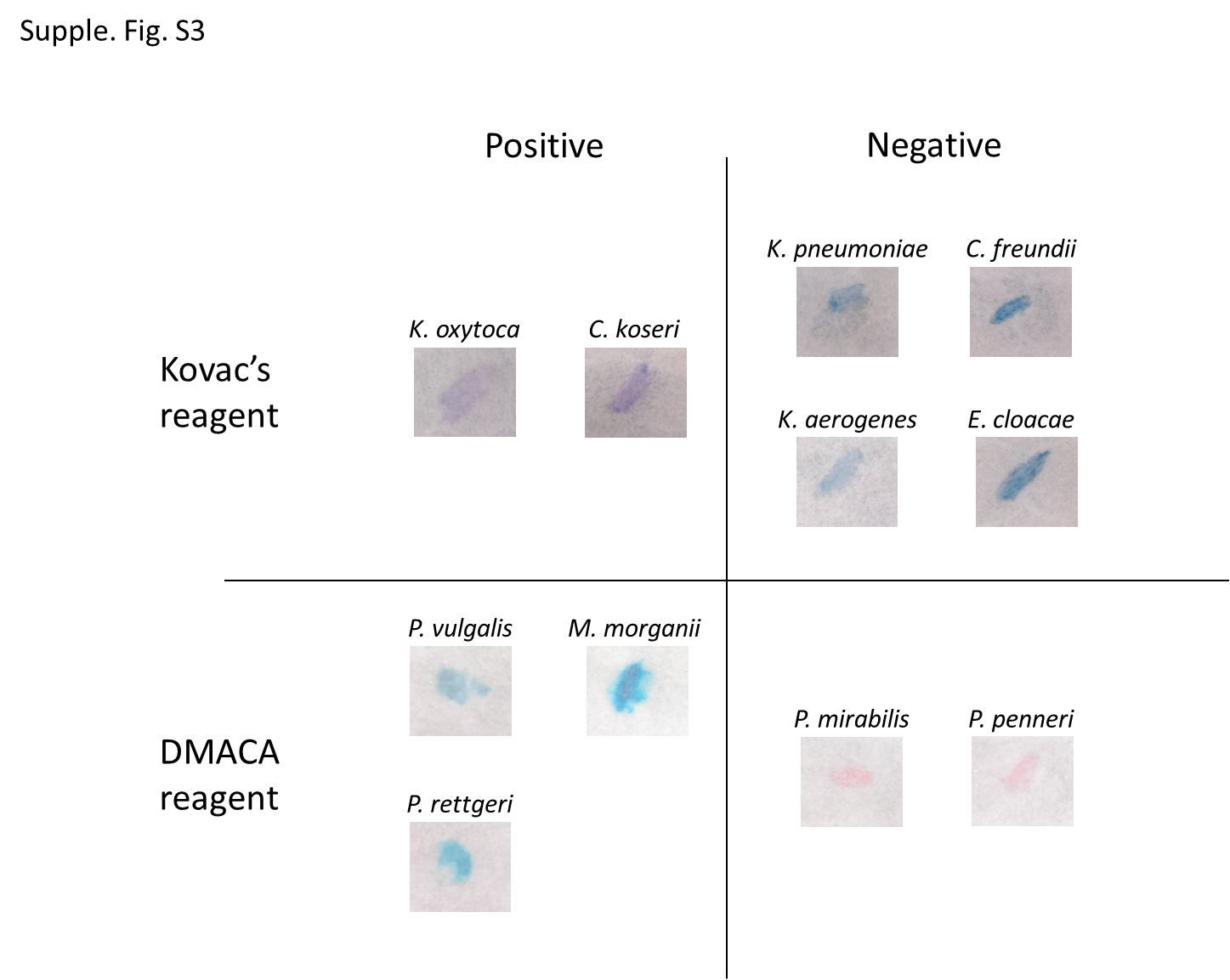

Supplement: Supplementary file 1 — Fig S1‐S3 [file JCLA-34-e23453-s001.docx]
